# Toxicity Assay for Citrinin, Zearalenone and Zearalenone-14-Sulfate Using the Nematode *Caenorhabditis elegans* as Model Organism

**DOI:** 10.3390/toxins10070284

**Published:** 2018-07-09

**Authors:** Julia Keller, Antje Borzekowski, Hajo Haase, Ralph Menzel, Liliane Rueß, Matthias Koch

**Affiliations:** 1 Department of Analytical Chemistry, Reference Materials, Bundesanstalt für Materialforschung und-Prüfung (BAM), Richard-Willstätter-Str. 11, 12489 Berlin, Germany; julia.keller@bam.de (J.K.); antje.borzekowski@bvl.bund.de (A.B.); 2 Department of Food Chemistry and Toxicology, Technische Universität Berlin, Gustav-Meyer-Allee 25, 13355 Berlin, Germany; haase@tu-berlin.de; 3 Institute of Biology, Ecology, Humboldt-Universität zu Berlin, Philippstr. 13, 10115 Berlin, Germany; ralph.menzel@biologie.hu-berlin.de (R.M.); liliane.ruess@biologie.hu-berlin.de (L.R.)

**Keywords:** mycotoxin, toxicity testing, food safety, *Caenorhabditis elegans*, metabolization

## Abstract

To keep pace with the rising number of detected mycotoxins, there is a growing need for fast and reliable toxicity tests to assess potential threats to food safety. Toxicity tests with the bacterial-feeding nematode *Caenorhabditis elegans* as the model organism are well established. In this study the *C. elegans* wildtype strain N2 (var. Bristol) was used to investigate the toxic effects of the food-relevant mycotoxins citrinin (CIT) and zearalenone-14-sulfate (ZEA-14-S) and zearalenone (ZEA) on different life cycle parameters including reproduction, thermal and oxidative stress resistance and lifespan. The metabolization of the mycotoxins by the nematodes in vivo was investigated using HPLC-MS/MS. ZEA was metabolized in vivo to the reduced isomers α-zearalenol (α-ZEL) and β-ZEL. ZEA-14-S was reduced to α-/β-ZEL-14-sulfate and CIT was metabolized to mono-hydroxylated CIT. All mycotoxins tested led to a significant decrease in the number of nematode offspring produced. ZEA and CIT displayed negative effects on stress tolerance levels and for CIT an additional shortening of the mean lifespan was observed. In the case of ZEA-14-S, however, the mean lifespan was prolonged. The presented study shows the applicability of *C. elegans* for toxicity testing of emerging food mycotoxins for the purpose of assigning potential health threats.

## 1. Introduction

**Figure 1:**
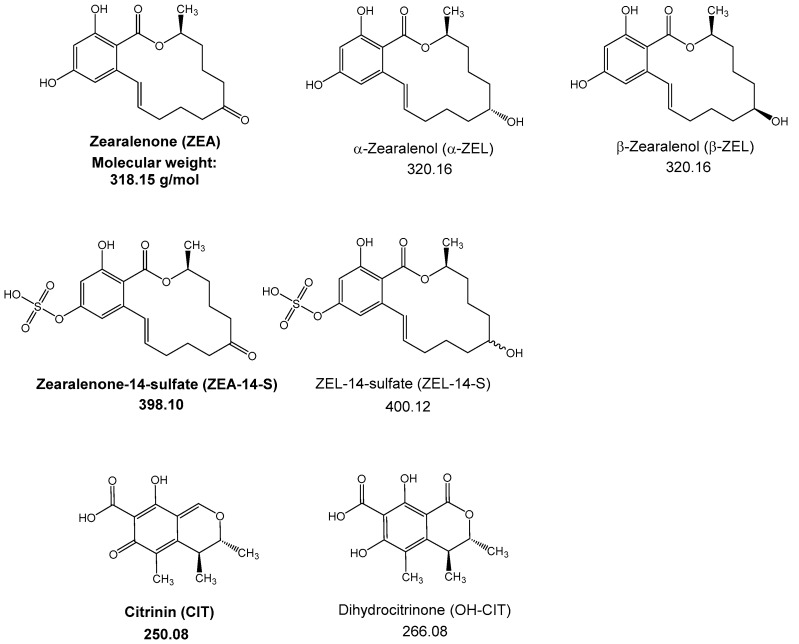
Chemical structures of the tested mycotoxins (shown in bold) and their related metabolites with their respective molecular weight.

Mycotoxins are secondary metabolites produced by a variety of filamentous fungi from across the world, contaminating approximately 25% of all harvested crops [1]. The intake of contaminated food and feed may lead to different kinds of acute and chronic diseases in humans and animals [2]. From 400–500 already known mycotoxins, only 11 are regulated and monitored by authorities within the EU [3,4]. Maximum levels have been set for zearalenone (ZEA) (Figure 1), which is mainly found in cereal crops such as maize, barley, wheat and rice, and is produced by several molds of the genus *Fusarium* [5,6]. ZEA induces estrogenic symptoms—such as uterine enlargement, vulvovaginitis or infertility—in higher animals such as rats, pigs or cows [7]. By causing DNA-adduct formation in in vitro cultures of bovine lymphocytes and in female mouse tissues ZEA has also been shown to act genotoxically [8,9]. Further toxic effects of ZEA are lipid peroxidation, cell death and the inhibition of protein and DNA synthesis [10]. In cultured pig, mouse and cattle, hepatocytes ZEA is metabolized by cytochrome P450 enzymes during phase I biotransformation to its reduced metabolites α-zearalenol (α-ZEL) and β-ZEL [11] (Figure 1). Using human and rat liver microsomes it has also been shown that ZEA may undergo oxidation to several mono-hydroxylated metabolites [12]. Furthermore, fungi and plants are able to modify ZEA by conjugation with sulfate or glucose at the aromatic OH-positions [13]. Mycotoxins and their modified forms are a cause of great concern for food safety and could pose a serious health risk due to their yet unknown toxic effects [14]. ZEA-14-sulfate (ZEA-14-S) (Figure 1) was originally found in cultures of *Fusarium graminearum* and is often detected in cereal-, soy- and corn-based products [15,16]. The nephro-, hepato- and cytotoxic mycotoxin citrinin (CIT) (Figure 1) is mainly found in stored grains [17]. Produced by molds of the *Penicillium*, *Monascus* and *Aspergillus* genus, CIT often co-occurs with the highly nephrotoxic and carcinogenic mycotoxin ochratoxin A and can be found in foodstuffs like breakfast cereals or rice [18,19,20]. According to the European Food Safety Authority (EFSA), there is still a substantial need for research to evaluate the occurrence and toxicity of CIT [21].

In order to evaluate the potential health risks posed by mycotoxins, in particular for emerging detected substances, reliable toxicity tests are necessary. Toxicity testing of mycotoxins is usually carried out by performing in vitro assays, or alternatively it is evaluated using laboratory animals like mice, rats or chickens in in vivo studies [22]. Since 1974 when Sydney Brenner described the cultivation and handling of *Caenorhabditis elegans*, this worm has been widely used as a model organism in developmental biology and neurology [23,24]. Due to its many advantages—such as its easy and cost-effective cultivation, the fact that the nematodes genome is completely sequenced and its short generation time—it also plays an growing role in toxicological research [25]. Finally, the high number of conserved genes between mammals and *C. elegans* make the worm an ideal candidate for toxicological investigations [26,27].

Surprisingly, to date, few studies are known to have used *C. elegans* to assess the toxic effects of mycotoxins. Yang et al. [28] described the multi-toxic endpoints of aflatoxin B_1_, deoxynivalenol (DON), fumonisin B_1_, T-2 toxin and ZEA using the *C. elegans* wildtype strain N2. They determined the lethality endpoint for ZEA with a LC_50_ of 75.79 mg/L and investigated alterations in lifespan and reproduction as well as morphological changes. The nematodes were all negatively affected by ZEA and the other tested mycotoxins [28]. Gowrinathan et al. [29] evaluated the toxicity of DON by performing partial and complete brood size assays using the *C. elegans* wildtype strain and a mutant strain (AU1) that has enhanced susceptibility to pathogens. The toxic effects of aflatoxin B_1_ in *C. elegans* were investigated by Feng et al. [30] by evaluating germline apoptosis, alteration of growth and reproduction. They found that the DNA damage response pathway was associated with the AFB_1_-induced germline apoptosis, which is highly relevant to reproductive and growth dysfunction in the worms.

Scientific studies in toxicological fields have improved the current knowledge of mycotoxins in food and feed. However, most concerns are linked to potential health risks of mycotoxins and their modified forms. Beside established toxicity tests with cell cultures or using mammalian models the toxicity testing with *C. elegans* can be an additional and valuable tool in mycotoxin research. Compared to toxicological experiments with cell cultures, assays with *C. elegans* provide data from a whole organism with intact and metabolically active systems [25]. In the present study we used *C. elegans* to investigate the toxic effects of the food-relevant mycotoxins CIT, ZEA and ZEA-14-S, on different life cycle parameters including reproduction, thermal and oxidative stress tolerance and lifespan. These are standard parameters in toxicological research with *C. elegans* and cover the most important toxic effects. Because no model is perfect, the assessment of multiple toxic endpoints could increase the sensitivity of the test and lead to more reliable results. Furthermore, for the first time, the metabolization of the mycotoxins in the worms was determined using HPLC-MS/MS to better understand the observed toxic effects in *C. elegans*.

## 2. Results and Discussion

To determine the toxicological impact of ZEA, ZEA-14-S and CIT on *C. elegans* as model organism different concentrations were tested. In case of CIT concentrations of 2.5 mg/L (10 µM), 12.5 mg/L (50 µM) and 62.5 mg/L (250 µM) were used according to findings from other studies [31,32]. For ZEA and ZEA-14-S concentrations of 7.5 mg/L (ZEA: 24 µM; ZEA-14-S: 19 µM) and 37.5 mg/L (ZEA:118 µM; ZEA-14-S: 95 µM) were used like described in Yang et al. [28] and adapted for the present study.

### 2.1. Metabolization of Mycotoxins In Vivo

**Figure 2:**
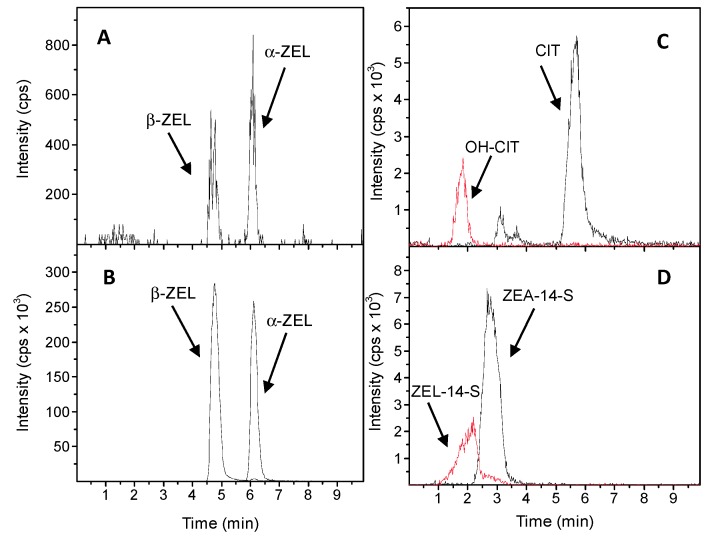
Worms fed for five days with mycotoxin containing OP50 bacteria were extracted and analyzed by HPLC-MS/MS in ESI negative mode using the multiple reaction mode with specific mass transitions given in material and methods. MRM chromatograms of worms treated with (**A**) zearalenone (37.5 mg/L) were compared with (**B**) a standard solution containing α-zearalenol and β-zearalenol. (**C**) citrinin (62.5 mg/L) and (**D**) zearalenone-14-sulfate (37.5 mg/L) are illustrated in black, whereas their metabolites are shown in red. Cps—counts per second.

After five days of cultivation on mycotoxin-enriched (CIT 62.5 mg/L, ZEA and ZEA-14-S 37.5 mg/L) and UV-inactivated feeding bacteria the worms were harvested, extracted and analyzed by HPLC-MS/MS (1100 series HPLC from Agilent Technologies, Waldbronn, Germany coupled to an API 4000 triple-quadrupole MS/MS system from Sciex, Framingham, MA, USA) in order to detect derived mycotoxin metabolites. The high conservation of genes and signaling pathways between mammals and *C. elegans* was shown in several studies and should result in the same or at least partially the same metabolic pattern of mycotoxins which have been found in mammalian models [25]. In the present study, ZEA was reduced in vivo by *C. elegans* to a lesser extent to α-ZEL and β-ZEL (Figure 2A) confirmed using standard solutions of α-ZEL and β-ZEL and the corresponding mass transitions (Figure 2B; residual ZEA not shown). The reduction of ZEA to its two stereoisomeric metabolites has already been widely described for mammals and is now shown in *C. elegans*, too [13,33]. Beside reductive metabolic reactions, also the oxidation of ZEA to its mono-hydroxylated metabolites was described by Pfeiffer et al. [34]. The formation of reduced and oxidized ZEA species is mainly catalyzed by several isoforms of cytochrome P450 enzymes. *C. elegans* has several CYP isoforms similar to those in higher animals and therefore the hydroxylation of ZEA is conceivable. However, during this study the analysis of extracted worms revealed no mono-hydroxylated ZEA metabolites or possible phase II metabolites like glucuronides.

Comparable to ZEA, the conjugated form ZEA-14-S was reduced to ZEL-14-S by *C. elegans* (Figure 2D). Due to the lack of a standard substance, currently no conclusion can be drawn about whether there are two stereoisomeric forms, and which stereoisomer it was that was metabolized. Surprisingly, in worms fed with ZEA-14-S no free ZEA was found, indicating that the worm is either not capable or only negligibly capable of deconjugating ZEA-14-S. In higher animals like pigs or in the deconjugation of ZEA-14-S in humans, the main biotransformation reaction is caused by bacteria in the colon during digestion processes by hydrolysis [13,35].

CIT was oxidized by *C. elegans* to its mono-hydroxylated form OH-CIT (Figure 2C). The major metabolite of CIT, OH-CIT, is found in human blood plasma and urine as a biomarker [36]. By testing the cytotoxic and genotoxic potential of OH-CIT compared to CIT, Föllman et al. [31] revealed that OH-CIT formation was a process of detoxification. It is conceivable that the key cellular metabolic pathways in *C. elegans* are to a certain extent conserved, leading to reduced and oxidized metabolites of mycotoxins which have been already described in mammalian models. Even if the metabolites are comparable, *C. elegans* lacks most mammalian organs and it would be not realistic to expect that *C. elegans* can be used to replace toxicological analyses in mammals. However, *C. elegans* as affordable and rapid model system could be a powerful tool in combination with other toxicity testing strategies and offers the opportunity to achieve relatively fast first toxicological data about emerging substances.

Beside the extracts derived from nematode biomass, the bacterial food solutions were extracted and measured with the same HPLC-MS/MS method. No metabolization of the added mycotoxin solution was detected (data not shown), indicating that the UV irradiation used effectively killed OP50 bacteria. This reveals UV irradiation to be an appropriate method for toxicity tests with bacterial consumers like *C. elegans*, as in contrast to killing using heat, UV light leaves the bacterial surface unaffected and unaltered. The use of heat to kill depresses the food quality of bacteria, potentially posing an additional stress to bacterial consumers, which can consequently distort toxicity testing.

### 2.2. Lifespan Assay

**Figure 3:**
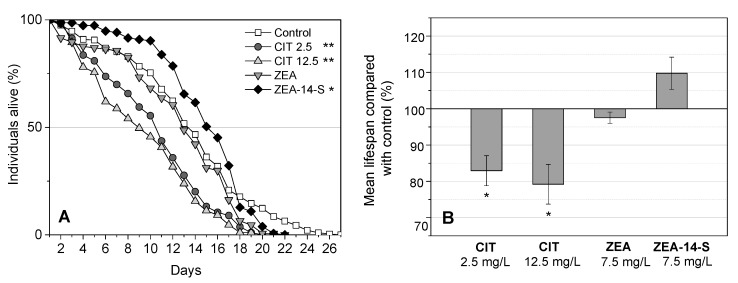
(**A**) Survival curves of *C. elegans* treated with single concentrations of citrinin (CIT 2.5 mg/L, 12.5 mg/L), zearalenone (ZEA 7.5 mg/L), zearalenone-14-sulfate (ZEA-14-S 7.5 mg/L) and untreated controls. Differences compared to the control were considered as significant with * *p* < 0.05 and ** *p* < 0.001 and were determined using the log-rank test; (**B**) The percentage change of mean lifespan compared to the control group during exposure to CIT, ZEA and ZEA-14-S. Each bar represents the mean of two to four trials with a total of at least 154 nematodes per concentration. (**A**,**B**) Error bars represent the standard error of the mean. Differences compared to the control were considered as significant with * *p* < 0.05.

A preliminary conclusion about the negative or positive effects of exogenous substances can be drawn by performing lifespan assays. According to the log-rank test, which is used to determine whether experimental treatments significantly altered the lifespan or not, ZEA-14-S significantly prolonged lifespan compared to the control group treated with dimethyl sulfoxide (DMSO), as shown in Figure 3A. The mean lifespan was extended by up to 9.8 ± 4.4% compared to the control group (Figure 3B). Several studies have described the life-prolonging effects of compounds in low doses that at higher concentrations induce toxic effects—the hormetic effect [37]. It is plausible that ZEA-14-S causes a mild stress in that low concentration range, leading to a slightly increased mean lifespan. The increased occurrence of deaths in the ZEA-14-S-treated group starting after 18 days (Figure 3A) may be due to the accumulation of the mycotoxin itself in the worm or of the derived metabolization products such as ZEL-14-S.

In Yang et al.’s study [28] treatment with ZEA using 10% of the estimated LC_50_ of ZEA, in this case 7.6 mg/L caused strong toxic effects, leading to a significant decrease in lifespan. However, the nearly same concentration of ZEA used in the present study did not cause a significant change in the mean lifespan compared to the control group but did lead to a shortened maximum lifespan (Figure 3A,B). Toxic effects may have accumulated over its lifetime and thus caused increased mortality of the test group after a certain point of time, in this case after 17 days of adulthood. However, in the Yang et al. study [28] 5-fluorodeoxyuridine (5-FUdR) was used to simplify the lifespan assay process. As side effects, in adult worms, 5-FUdR slightly reduced the pharyngeal pumping rate, produced changes in body size and morphology, and increased superoxide dismutase levels [38]. The strong, toxic effects observed by Yang et al. [28] may have been caused by the simultaneous use of two substances, the toxin and the progeny blocker 5-FUdR.

Both concentrations of CIT tested had a significant negative effect on the lifespan of *C. elegans* (Figure 3A). The highest concentration of citrinin with 12.5 mg/L led to an increased mortality rate compared to the test group with 2.5 mg/L of citrinin. The mean lifespan was decreased to 11.5 ± 1.3 days (CIT 2.5 mg/L) and 11.0 ± 1.5 days (CIT 12.5 mg/L) which is a reduction of 17.1 ± 4.1% and 20.8 ± 5.5% compared to the control group (Figure 3B). The influence the CIT’s concentration has on the alteration of lifespan is greater during the first ten days of adulthood. This concentration dependence nearly vanished after 11 days and the lifespan curves ran similarly from that point on. It is conceivable, that it was the conversion of CIT to the less toxic mono-hydroxylated CIT, observed by HPLC-MS/MS measurements that caused this effect.

### 2.3. Brood Size Assay

**Figure 4:**
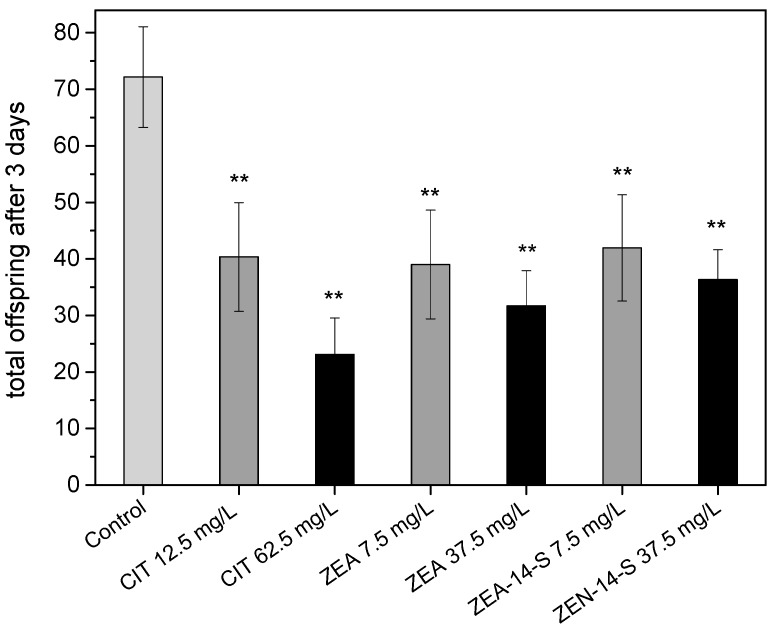
Effects of mycotoxins on the brood size of *C. elegans* after three days of reproduction, determined with at least 26 animals per concentration, displayed as the average of three trials; error bars represent the standard error of the mean. Differences compared to the control were considered as significant with ** *p* < 0.001.

The average number of offspring after three days was significantly reduced at all tested concentrations (Figure 4). Compared to the number of offspring of the control group, with 72.2 ± 8.9 larvae, CIT decreased the number of nematode offspring produced significantly to 40.4 ± 9.6 (12.5 mg/L) and 23.1 ± 6.4 (62.5 mg/L). Among other toxic effects CIT has also been found to negatively affect the reproductive system. In Institute of Cancer Research (ICR) mice 2.5 and 5.0 µM of CIT reduced the oocyte maturation rate, as well as fertilization and embryo development [39]. Furthermore, female rats that were mated with CIT-treated males displayed a reduced pregnancy rate, as shown by Qingqing et al. [40]. 

Treatment with ZEA led to a reduction in the number of offspring to 39.0 ± 9.6 (7.5 mg/L) and 31.7 ± 6.1 by using 37.5 mg/L of ZEA, respectively. ZEA is known to cause various estrogenic effects in laboratory and domestic animals such as decreased fertility or alterations in the reproductive tract, whereby pigs and sheep appear to be more significantly affected by ZEA than rodents [33]. The influence of mycotoxins on reproductive fitness had also been previously shown by Yang et al. who observed negative effects on the N2 nematode’s reproduction in the case of fumonisin B_1,_ aflatoxin B_1_, T-2 toxin and ZEA. Toxic effects on reproduction were more pronounced with higher concentration in all three tested mycotoxins, as shown in Figure 4. ZEA and ZEA-14-S had comparable toxic effects on reproduction. To date, only a few studies have investigated the toxic effects of conjugated mycotoxin forms, although modified toxins represent an emerging problem. Conjugation with glucose or sulfate is a known detoxification process in plants and it is supposed that conjugated mycotoxins possess a lower toxicity compared to their parental compounds. However, in this study, ZEA-14-S reduced the number of offspring to an average of 41.9 ± 9.4 (7.5 mg/L) and 36.3 ± 5.3 (37.5 mg/L) individuals.

The comparatively strong effect of ZEA-14-S in the reproduction assay is remarkable (Figure 4), particularly in light of its dramatically decreased estrogenicity at the estrogen receptor level compared to ZEA [41]. As an *r*-selected species *C. elegans* starts reproduction early, and a lowered progeny could result in the reduction or even the extinction of a population as reproductive fitness is a crucial factor for maintaining population size. To sum up, *C. elegans* appears to be an appropriate candidate for testing the reproductive toxicity of mycotoxins.

### 2.4. Oxidative and Thermal Stress Resistance Assay

**Figure 5:**
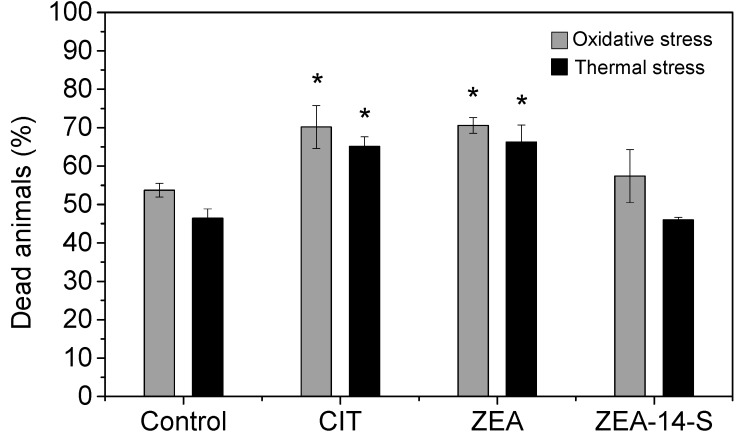
Effect of 62.5 mg/L citrinin (CIT), 37.5 mg/L zearalenone (ZEA) and 37.5 mg/L zearalenone-14-sulfate (ZEA-14-S) on the oxidative and thermal stress resistance of *C. elegans*. On the sixth day of adulthood, alive and dead animals were counted after a treatment of 8 h at 35 °C for the thermal stress resistance assay (black bars) and after an 8 h exposure to 0.8 mM hydrogen peroxide for the oxidative stress resistance assay (grey bars). At least 174 nematodes per mycotoxin were scored for the thermal stress assay and 122 for the oxidative stress assay. Bars show the average of all three trials and the error bars represent the standard error of the mean. Differences compared to the control were considered as significant with * *p* < 0.05.

The treatment of *C. elegans* with CIT and ZEA led to a significantly increased mortality rate after 8 h of oxidative stress to 70.2 ± 5.6% (CIT) and 70.6 ± 2.1% (ZEA), compared to the control group with 53.7 ± 1.8%, as shown in Figure 5. Pascual-Ahuir et al. [42] investigated the mechanisms of CIT toxicity using the quantitative yeast model and observed the cellular defense mechanism in response to the mycotoxin. They concluded that CIT triggered the activation of stress responsive promoters like the glucocorticoid response element 2 (GRE2) and superoxide dismutase 2 (SOD2). Consequently, the induction of reactive oxygen species (ROS) could be the predominant toxicity mechanism of CIT. Hassen et al. [43] investigated the role of oxidative stress in ZEA-mediated toxicity by performing test assays with human HepG2 cells. This cell type responded to ZEA treatment with a loss of cell viability and the induction of oxidative DNA damage. Findings in the present study indicated that the ZEA-induced toxicity in *C. elegans* could be related to oxidative damage in the worms. For both mycotoxins there are indications that toxic effects in the worms could be probably driven by stress responsive factors. As a result, during this specific assay, these toxins perhaps negatively affected the stress resistance of *C. elegans* and thus led to enhanced mortality. Under thermal induced stress, N2 wildtype nematodes treated with CIT (62.5 mg/L) and ZEA (37.5 mg/L) showed higher mortality rates compared to the untreated control group (and ZEA-14-S). However, studies concerning the modes of toxicity caused by ZEA-14-S are still lacking. Several studies have found that in *C. elegans* longevity and stress resistance are linked [44]. Thus, it might be surprising that after having slightly positive effects on lifespan, ZEA-14-S failed to induce statistically significant changes in either the thermal stress assay or the oxidative stress assay. However, not all long-lived nematodes are more stress resistant and Cypser et al. [45] even proposed that thermal stress resistance and lifespan alteration were triggered by different molecular signals [46,47].

## 3. Conclusions

To date, toxicological data for modified mycotoxins like ZEA-14-S or emerging mycotoxins like CIT are scarce or even non-existent. The model organism *C. elegans* that can provide a bridge between conventional in vitro and in vivo assays using mammalian models was used in this study as a simple, high throughput and cost-effective method to evaluate the toxicity of selected mycotoxins. Only a few toxicological studies on modified mycotoxins have already been carried out and reveal deconjugation to free mycotoxins as a preferred pathway of biotransformation. However, data obtained in this study showed the reduction of ZEA-14-S to ZEL-14-S in *C. elegans*. Probably due to the highly conserved genes and metabolic pathways between *C. elegans* and mammals, the metabolization of ZEA to its reduced and more estrogenic isomers α-ZEL and β-ZEL was found, as well as the mono-hydroxylation of CIT. There is still a considerable need for further research concerning the metabolic fate and toxicity of food-relevant mycotoxins. In combination with identification techniques such as HPLC-MS/MS and high-resolution mass spectrometry or other toxicological model systems *C. elegans* is an ideal candidate for first toxicity screenings, not only in mycotoxin research.

## 4. Materials and Methods

### 4.1. Chemicals and Substances

Citrinin was purchased from Fermentek (Jerusalem, Israel) with a purity of 98%. Zearalenone was purchased from SantaCruz Biotechnology Inc. (Santa Cruz, CA, USA) with a purity of 99%. Zearalenone-14-sulfate was produced by a double-staged biosynthesis and preparative fractionation as previously described [48]. α-zearalenol (α-ZEL), β-ZEL and dimethyl sulfoxide were purchased from Sigma-Aldrich Chemie GmbH (Steinheim, Germany). All standard chemicals were of p.a. grade, and all solvents of HPLC grade. Nematode growth media (NGM) powder was purchased from USBio (Salem, MA, USA) and lysogeny broth (LB) medium from Merck KGaA (Darmstadt, Germany). One liter of NGM medium was prepared as recommended by the manufacturer by adding 25 mL of 1M phosphate buffer (pH 6), 1 mL of 1 M CaCl_2_ and 1 M MgSO_4_ (both purchased from Merck KGaA; Darmstadt, Germany). Ultrapure water was produced using a Seralpur PRO 90 CN system (Seral Reinstwassersysteme GmbH, Ransbach-Baumbach, Germany).

### 4.2. Strains and Conditions

The *C. elegans* wildtype strain N2 was used and maintained at 20 °C on sterile NMG media. As a food source, *Escherichia coli* OP50 bacteria were used according to Brenner, 1974 [23]. The wildtype strain N2 and OP50 bacteria were kindly derived by the Caenorhabditis Genetics Center (University of Minnesota). For the cultivation of OP50 bacteria one colony was picked from an LB agar plate and brought in liquid LB medium and was shaken slightly over night at 37 °C. To kill the bacteria, sterilized quartz vessels were filled with 20 mL of the freshly prepared culture and irradiated by UV-C light at *λ* = 254 nm for 2 h by gentle shaking. The inactivation of bacteria was tested by plating them on LB agar and incubating them overnight at 37 °C; no bacteria growth was observed. Mycotoxins were added to the UV-killed bacteria to obtain final concentrations between 2.5 mg/L and 62.5 mg/L. Equal amounts of test solutions were used for all conditions (final concentration of 0.3% [*v*/*v*] DMSO) and control solutions were mixed with DMSO.

### 4.3. Lifespan Assay

For each treatment 20–30 L4 larvae—easily distinguishable by the presence of a small, white half-circle patch in the worm midsection—were transferred with a small platinum wire to 70 × 15 mm petri dishes with 1 mL of mycotoxin containing bacteria and cultivated at 20 °C. From the following generation (F1), 150 L4 larvae were transferred to five small plates (35 × 12 mm) with 15 individuals per plate representing one trial. Two to four trials were performed per concentration. The number of surviving and dead animals were counted every day and the dead animals were removed from the plate. Individuals that died from internal hatching or that crawled out of the NMG medium were considered as being neither dead or alive and were excluded from the assay. Animals that failed to react after being gently touched with the platinum wire were rated as dead. During their reproductive phase (approx. 5 days) nematodes were transferred daily to avoid starvation or the mixing of generations and were fed with 150 µL of bacteria. After that, worms were transferred every two to three days. Plates contaminated with bacteria or molds were dismissed from the trial. Statistical significance for alteration of the mean lifespan was calculated using the log-rank test provided online by the Bioinformatics at the Walter and Eliza Hall Institute of Medical Research (http://bioinf.wehi.edu.au/software/russell/logrank).

### 4.4. Brood Size Assay

L4 larvae (F1) were separated on small petri dishes and the offspring of each worm was counted after three days of adulthood. The total number of offspring was determined, and the statistical significance was calculated using One-way ANOVA. Two to three trials per condition and control were performed, with at least 20 animals per trial.

### 4.5. Thermal and Oxidative Stress Resistance Assay

For every concentration and trial about 20–30 L4 larvae (F1) were used. On the sixth day of adulthood, untreated and treated animals were moved to 35 °C incubation for 8 h and the number of dead and surviving nematodes were counted for the thermal stress resistance assay. For the oxidative stress resistance assay nematodes were transferred to M9 buffer containing 0.8 mM hydrogen peroxide and were incubated at 20 °C for 8 h. Nematodes were gently touched with a platinum wire. If they failed to respond, they were counted as dead. The statistical significance was calculated by using One-way ANOVA.

### 4.6. Cultivation and Extraction of Nematodes for Metabolite Analysis 

*Cultivation* Mixed cultures were grown on NGM agar on 60 big petri dishes. To avoid starvation OP50 mixed with the test substances was provided during cultivation at 25 °C at least once a day. After five days of cultivation nematodes were rinsed from the plates using ice-cold M9 buffer, collected in 50 mL tubes and stored for the duration on ice. Worms were washed three times with 10 mL of M9 buffer to remove bacteria. In between, worms were allowed to settle to the bottom; afterwards the supernatant was always removed from the worm pellet. The final pellets were kept frozen at −80 °C for further extraction and analysis.

*Extraction* About 500 µL of worm pellet was dissolved with 4.5 mL of acetonitrile-water mixture (80:20 *v*/*v*) and treated for 10 min in an ultrasonic bath. After subsequent shaking with a horizontal shaker for 30 min at 330 min^−1^, samples were centrifuged for 10 min at 2931× *g* at 20 °C. For further analysis, 1 mL of the supernatant was evaporated under nitrogen at 40 °C and re-dissolved in HPLC eluent (65:35 *v*/*v* acetonitrile-water with 0.1% formic acid). For the extraction of OP50 bacteria an overnight culture was UV-killed and mixed with mycotoxin dissolved in DMSO (or only pure DMSO as control) and stored for 5 days at 4 °C to simulate the maximal usage time for a bacteria solution. About 10 mL of the bacteria culture was centrifuged for 10 min at 3828× *g*. The supernatant was kept and stored at −80 °C and the bacteria pellet was washed twice with M9 buffer. The extraction of the supernatant and bacteria was performed in the same way as the extraction of the worms.

### 4.7. HPLC–MS/MS Analysis

HPLC–MS/MS measurements were performed using a 1100 series HPLC from Agilent Technologies (Waldbronn, Germany) coupled to an API 4000 triple-quadrupole MS/MS system (Sciex, Framingham, MA, USA). A Synergi Polar–RP column (150 mm × 3 mm; particle size: 4 µm; pore size: 80 Å) from Phenomenex (Torrance, CA, USA) was used. The column oven was set to 40 °C and the solvents used were water with 0.1% formic acid (solvent A) and acetonitrile with 0.1% formic acid (solvent B) using a flow rate of 800 µL/min. The gradient was as follows: 0–1 min isocratic with 35% B, 1–29 min linear to 100% B, isocratic 29–34 min 100% B, shifting back to 35% B and reconditioning from 35–40 min. The injection volume used was 20 µL and the ESI was operated in the negative ionization mode at 450 °C. The settings were as follows:

Curtain gas 20 psi, collision gas 8 psi, ion source gas 1 and 2 with 60 psi, respectively, and an ionization voltage of −4500 V. The MS/MS measurements were performed in the multiple reaction monitoring (MRM) mode. Mass transitions were recorded as follows for the investigated analytes by using a declustering potential of −60 V and a collision energy of −30 eV: ZEA *m*/*z* 317.1 → quantifier *m*/*z* 130.1/qualifier *m*/*z* 174.8; ZEL *m*/*z* 319.2 → 174.0/160.0; ZEA-14-S *m*/*z* 397.1 → 317.1/175.0; ZEL-14-S *m*/*z* 399.2 → 319.2/275.2; CIT *m*/*z* 249.0 → 205.0, OH-CIT *m*/*z* 265.0 → 221.0.
